# Effects of Plantar Vibration on Bone and Deep Fascia in a Rat Hindlimb Unloading Model of Disuse

**DOI:** 10.3389/fphys.2018.00616

**Published:** 2018-05-23

**Authors:** Yunfei Huang, Yubo Fan, Michele Salanova, Xiao Yang, Lianwen Sun, Dieter Blottner

**Affiliations:** ^1^Key Laboratory for Biomechanics and Mechanobiology of Ministry of Education, School of Biological Science and Medical Engineering, Beihang University, Beijing, China; ^2^Beijing Advanced Innovation Centre for Biomedical Engineering, Beihang University, Beijing, China; ^3^Institute of Vegetative Anatomy, Charité – University Medicine Berlin, Berlin, Germany; ^4^Center of Space Medicine Berlin, Neuromuscular Group, Charité – University Medicine Berlin, Berlin, Germany

**Keywords:** bone, deep fascia, hindlimb unloading, biomechanics, vibration exercise, collagen, extracellular matrix

## Abstract

The deep fascia of the vertebrate body comprises a biomechanically unique connective cell and tissue layer with integrative functions to support global and regional strain, tension, and even muscle force during motion and performance control. However, limited information is available on deep fascia in relation to bone in disuse. We used rat hindlimb unloading as a model of disuse (21 days of hindlimb unloading) to study biomechanical property as well as cell and tissue changes to deep fascia and bone unloading. Rats were randomly divided into three groups (*n* = 8, each): hindlimb unloading (HU), HU + vibration (HUV), and cage-control (CON). The HUV group received local vibration applied to the plantar of both hind paws. Micro-computed tomography analyzed decreased bone mineral density (BMD) of vertebra, tibia, and femur in HU vs. CON. Biomechanical parameters (elastic modulus, max stress, yield stress) of spinal and crural fascia in HU were always increased vs. CON. Vibration in HUV only counteracted HU-induced tibia bone loss and crural fascia mechanical changes but failed to show comparable changes in the vertebra and spinal fascia on lumbar back. Tissue and cell morphometry (size and cell nuclear density), immunomarker intensity levels of anti-collagen-I and III, probed on fascia cryosections well correlated with biomechanical changes suggesting crural fascia a prime target for plantar vibration mechano-stimulation in the HU rat. We conclude that the regular biomechanical characteristics as well as tissue and cell properties in crural fascia and quality of tibia bone (BMD) were preserved by local plantar vibration in disuse suggesting common mechanisms in fascia and bone adaptation to local mechanovibration stimulation following hind limb unloading in the HUV rat.

## Introduction

Research from space and ground based analog studies (bed rest) showed that microgravity and chronic disuse induced unloading causes bone loss with muscle atrophy (Vico et al., 2000; Pavy-Le Traon et al., 2007). Notably, significant bone loss in astronauts and in bed rest participants was mainly found in the lower limbs but also in the spine (Vico et al., 2000; Dana Carpenter et al., 2010). However, exercise countermeasures presently adopted in space have only limited success (Korth, 2015; Sibonga et al., 2015) suggesting optimization of countermeasure prescriptions for the benefit of successful future long-term spaceflight missions. A more effective and less time-consuming exercise training against muscle and bone loss and related tendon and fascia structures is therefore required to minimize the negative effects of spaceflight to the astronauts’ musculoskeletal and myofascial system and to also improve health management in terrestrial sedentary populations as well as in various clinical settings (Lang et al., 2017).

Animal models of disuse and human analog studies are well-established on the ground to study the effects and outcomes of different countermeasures to ameliorate if not prevent deconditioning of the musculoskeletal system. WBV was applied to prevent the decrease in femoral strength and bone loss in hindlimb unloading rats (Rubin et al., 2001). In addition, local vibration (35 Hz, 2.4 g) was successfully applied in the same rat model of disuse (Sun et al., 2014). However, studies also showed that WBV did not prevent from an increase in bone resorption and the effects of bed rest on bone (Baecker et al., 2012). The WBV (30 Hz) with a lower acceleration was found to show little or no effects on ovariectomy-induced bone loss in a comparative study of different acceleration (0.6 g, 3 g) (Rubinacci et al., 2008). Russo’s study found that a 0.1–10g, 28 Hz vibration was useless in preventing bone loss of postmenopausal women and even worse than control (Russo et al., 2003). WBV is thought somehow to damage the peripheral vessel of animals and, for example, make people feeling uncomfortable during and after WBV exposition (Murfee et al., 2005; Kiiski et al., 2008). The inconsistent results of vibration reported so far suggested that either amplitude, frequency, or even duration of vibration protocols could well have influenced outcome for example bone loss. Anyway, vibration exercise as countermeasure showed promising outcome in the structural and functional preservation particularly addressing calf muscle and bone outcome in otherwise healthy male long-term bed rest participants (Blottner et al., 2006; Rittweger et al., 2010). In general, vibration stimulation was able to enhance trabecular bone formation, increase the expression of bone formation related genes, reduce the osteoclast activity, and inhibit bone loss in the spine and femur (Cardinale and Bosco, 2003; Castrogiovanni et al., 2016). In the present work, we therefore tested if local vibration applied to more appropriate anatomical sites such as the plantar region of the hind paw might be an alternative strategy to more efficiently prevent loss in bone mineral density and maladaptation to the associated myofascial tissue in a rat model of disuse.

Besides bone, muscle and tendon, the deep fascia of the vertebrate body comprises a biomechanically unique connective cell and tissue layer with integrative functions to support global and regional strain, tension, and even muscle force during movement and performance control. An estimated 30–40% of the total muscle force and power is likely stored by the fascia and its mainly collagenous 3-dimensional fiber network linked to peculiar viscoelastic extracellular matrix components (Huijing and Jaspers, 2005; Huijing, 2009; Turrina et al., 2013). With the adherence of muscle and fascia, force is transmitted multi-directionally in both longitudinal and transversal orientation through intramuscular connective tissues (endomysium, perimysium, epimysium), and even further through the deep fascia layers to tendons and their bone insertions (Huijing and Jaspers, 2005). We focussed our present research on the targeted effects of plantar vibration to maintain key biomechanical properties of deep fascia and to hopefully defend against a suspected disproportionate remodeling of collagenous network and functional cells in the deep fascia tissue layers following disuse.

The main study objectives were to investigate changes in cellular, tissue and biomechanical properties of deep fascia in relation to the bone quality in a rat HU as a model of disuse with and without plantar vibration mechanosignals. We used a custom-made vibrating bipedal device that alternately generates local plantar vibration targeted directly to the rat’s hind paw in disuse (Sun et al., 2014).

## Materials and Methods

### Experimental Animal Groups and General Tissue Sampling Procedure

All animal treatments were approved by the Animal Care Committee of Peking University in accordance with the Regulation of Administration of Affairs Concerning Experimental Animals of State Science and Technology Commission of China. Twenty-four (*n* = 24) female 8-week-old Sprague-Dawley rats were recruited from the Experimental Animal Center of Peking University, China. After 7 days of adaptation, the rats were randomly divided into three groups (*n* = 8, each group): HU, HUV, and CON. The rats hindlimb in HU and HUV group were unloading for 21 days as previously described (Morey-Holton and Globus, 2002). Each single rat in three groups was housed separately in the same cages in the animal facility of the laboratory with lab chow and water ad libitum. Room temperature was controlled at 25 ± 2°C with a 12/12 h light/dark cycle. After 21 days, the rats were sacrificed by narcotic overdose (1% Pentobarbital Sodium, 18 ml/kg, intraperitoneally). After removal of the skin, the right part of lumbar back and crural fascia tissue layers were dissected separately from muscle and preserved in PBS at 4°C for mechanical testing (see below). The left parts of lumbar and crural fascia were dissected with skeletal muscle attached, immediately frozen in liquid nitrogen, and finally cryopreserved at -80°C for qualitative and semiquantitative immunohistochemistry (see below). Finally the tibiae, femora, and vertebra bones were harvested and scanned by μCT according to previously described protocols (Sun et al., 2014).

### Hindlimb Unloading (Model of Disuse)

Hindlimb unloading was achieved as previously established by Morey-Holton and Globus (2002). The three groups of rats, one without (HU, *n* = 8), one with plantar vibration exercise (HUV, *n* = 8), and one cage control group (normal cage habitats, no HU, *n* = 8) were used for comparison. Briefly speaking, the rat’s tail was cleaned with 70% ethanol and sprayed with benzoin and rosin tinctures to increase the adhesiveness. When it was dried, the tail was suspended by two tail-parallel strips of adhesive tape attached. They were protected with three strips of Gauze bandage to prevent the tail peeling off the tape. Then the adhesive tape was tied to a fish-line swivel that hanged on a chain from the top of the cage (30 cm × 30 mm × 50 cm) allowed rats move freely on a *x*–*y* axis and rotate 360°. The tape and bandage were changed every 7 days considering the growing size of the tail. The body was maintained in HU rats at approximately 30° angle from the cage floor (hind limbs up avoiding cage floor contact) while animals were allowed to move freely with their front paws on the cage floor to have access to food and water ad libitum.

### Plantar Vibration

**FIGURE 1:**
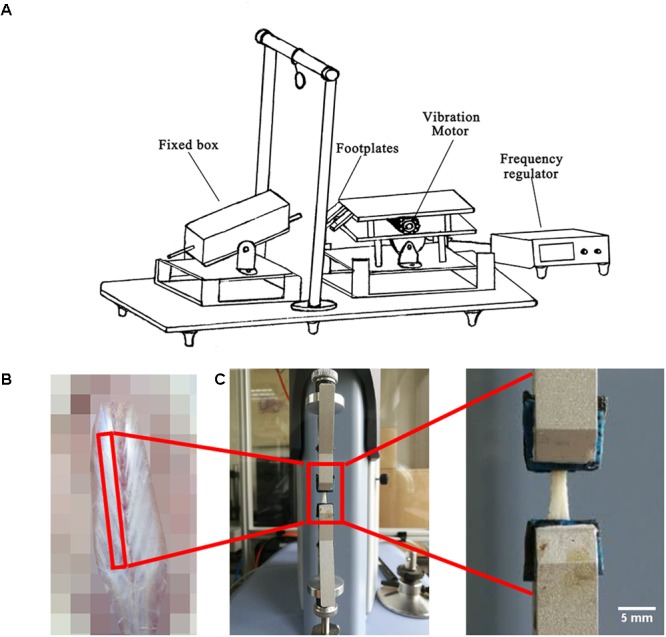
Experimental set-up, fascia preparation and mechanical tests. **(A)** During hindlimb unloading plus local vibration (HUV) training sessions (*n* = 8 rats) hind claws are fixed by adhesive tape to vibrating foot plates (alternate acceleration, 200 s/35 Hz/1 mm amplitude, twice a day for 21 days). **(B)** Spinal fascia preparation outlined (skin removed). **(C)** Mechanical test device with clipped fascia preparation (arrow). Bar represents 5 mm.

Local vibration in HUV was applied to the planta of each rat hind paws twice a day (at 8 a.m. and 5 p.m. for 200 s per session) for 21 days using a special custom made vibration device previously reported (Sun et al., 2014; **Figure 1A**). During vibration, the HU rat’s body was always maintained at -30° angle in a fixed box. During exercise sessions, the hind paws were immobilized on the bipedal footplates of the device with medical adhesive tape. An electronically controlled motor connected to an eccentric bearing generated the vibration at constant settings (35 Hz, 1 mm amplitude, 2.4 g acceleration). Animals in the HU group were sham-loaded in the device without vibration.

### Bone Mineral Density by μCT

Bone mineral density of vertebra L3, distal femur and proximal tibia were measured by μCT (SkyScan 1076, Belgium) as previously reported (Sun et al., 2014). Scanning parameters were as follows: 70 kV voltage, 140 μA current, 1 mm aluminum filter, 18 μm pixel size, 180° rotation and 0.6° step. Reconstruction parameters were one smoothing, eight ring artifacts and 30% beam hardening. An 1898 μm-thick trabecular region (100 reconstruction images), 1898 μm distance from the growth plate was analyzed in the distal femur or proximal tibia. An 1898 μm-thick trabecular region in the middle of L3 vertebrae was analyzed. The region of interest in each image was delineated automatically by plug-ins of the software CTAn (v.1.14.4, SkyScan, Belgium).

### Fascia Mechanical Tests

A 4 cm-long strip of freshly dissected lumbar spinal fascia (**Figure 1B**) and a 2 cm-long strip of crural fascia were both immersed in PBS and fixed on a rectangle little piece of wood with very thin inert pins to maintain their original shapes. The crural fascia ensheaths the plantar flexor triceps surae that inserts to the calcaneus bone by the Achilles tendon. All fascia samples were further preserved in PBS at 4°C several hours. Afterwards, the PBS immersed fascia was cut in a rectangular shape to facilitate fast fixing of the PBS soaked fascia strips to the small clips of the mechanical testing device. The biomechanical properties (*n* = 8) were measured through tensile test (Shimadzu EZ-LX, Japan) using a 50 N mechanical sensor, 2 mm/min loading, and one tensile loading to break (**Figure 1C**). Through the tensile test, the force-displacement curve with all raw data was obtained. The height, width and thickness of the fascia were measured by vernier caliper. Stress = force/(width × thickness). Strain = displacement/height. Then the stress-strain curve was drawn. Elastic modulus is the slope of stress-strain curve in the elastic deformation region. Max stress (also called ultimate tensile strength) is the stress value of the highest point of the stress-strain curve. Ultimate strain is the strain value of the highest point of the stress-strain curve. Yield stress is the stress value of the end point of the elastic deformation region in the stress-strain curve. Yield strain is the strain value of the end point of the elastic deformation region in the stress-strain curve.

### Immunohistochemistry

Identical fascia strips from the left side of the back and calf muscle were prepared and immediately fixed on a piece of wood without getting dry as described above. Then fascia preparations were immediately frozen in liquid nitrogen and stored at -80°C. Series of cryosections (thickness = 8 μm) of fascia were cut (LEICA Cryocut 1800). The normal histology of the CON rat crural fascia is shown in cross-cut cryosections by two routine staining protocols in light microscopy. After methylene blue and HE staining, the integrated sections from each group (*n* = 5, each) were chosen for immunofluorescence staining.

They were immunostained with collagen I, collagen III, and matrix metalloproteinases-2 (MMP-2) antibodies according to the following protocols: The cryosections mounted on protein-coated glass slides (Superfrost, Germany) were postfixed with 4% paraformaldehyde for 10 min at 4°C. Samples were permeabilized with PBS with 2% bovine serum albumin (BSA) with 0.3% triton X-100 (Sigma, United States) for 1 h at room temperature. As primary antibodies we used a goat polyclonal antibody to collagen I (1:20 dilution, Santa Cruz Biotechnology, United States), a mouse monoclonal antibody to collagen III (1:500 dilution, Sigma, United States), and rabbit polyclonal antibody to MMP-2 (1:100 dilution, Santa Cruz Biotechnology, United States). After primary antibody incubation, sections were next incubated with secondary antibodies using donkey anti-goat Alexa 488 (1:1000 dilution) and goat anti-mouse Alexa 488 (1:1000 dilution), and goat anti-rabbit Alexa 488 (1:1000 dilution). The cell nuclei were immunostained with fluorescent DAPI (Vector, United States) in soluble embedding medium (Vector, United States).

Images were obtained with a confocal laser scanning microscope (Leica TCS SP2; Leica Microsystems, Bensheim, Germany). Immunofluorescence intensity of collagen I, collagen III and MMP-2 in specific areas on the tissue sections was quantified and expressed as arbitrary units (a.u.) per pixel-area intensity in a range of 0–255 using the Leica software (LAS X). Nuclear density, fascia thickness in collagen I stained sections, and area and number of collagen III bundles were analyzed in at least three separate tissue sections each using ImageJ free software (NIH, United States).

### Statistical Analysis

Statistical analyses were performed with SPSS 20.0 using non-parametric analyses with independent-samples Kruskal–Wallis One Way ANOVA test followed by all pairwise multiple comparisons (Dunn-Bonferroni *post hoc*). All values were expressed as means ± standard deviation. The level of statistical significance was set at *P* < 0.05.

## Results

### Biomechanical Properties of Bone and Deep Fascia

**FIGURE 2:**
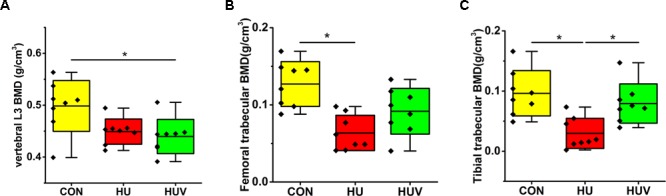
Bone mineral density (BMD) analyses by μCT in all rat groups (each, *n* = 8). **(A)** Lumbar vertebra L3 with moderate BMD loss in HUV vs. cage-control (CON). **(B)** Femur with BMD loss in both hindlimb unloading (HU) (–49.95%) and HUV (–27.80%, non-significant) vs. CON. **(C)** Tibia with strong BMD loss in HU (–69.18%) and no loss in HUV vs. CON. All groups are plotted as means = single horizontal black line in color bar; +/– standard deviation = top/bottom line of the box; max/min values = top/bottom of the bar line; Individual values were superimposed as dots. ^∗^*P* < 0.05.

A moderate loss in BMD was found in L3 vertebrae (-9.93%, non-significant), and to a larger extent in both femur (-49.95%) and tibia (-69.18%) in HU rats compared to CON rats (**Figure 2**). However, BMD values in HUV rats were found to be unchanged in tibia bone vs. CON (**Figure 2**).

#### Spinal Fascia

**FIGURE 3:**
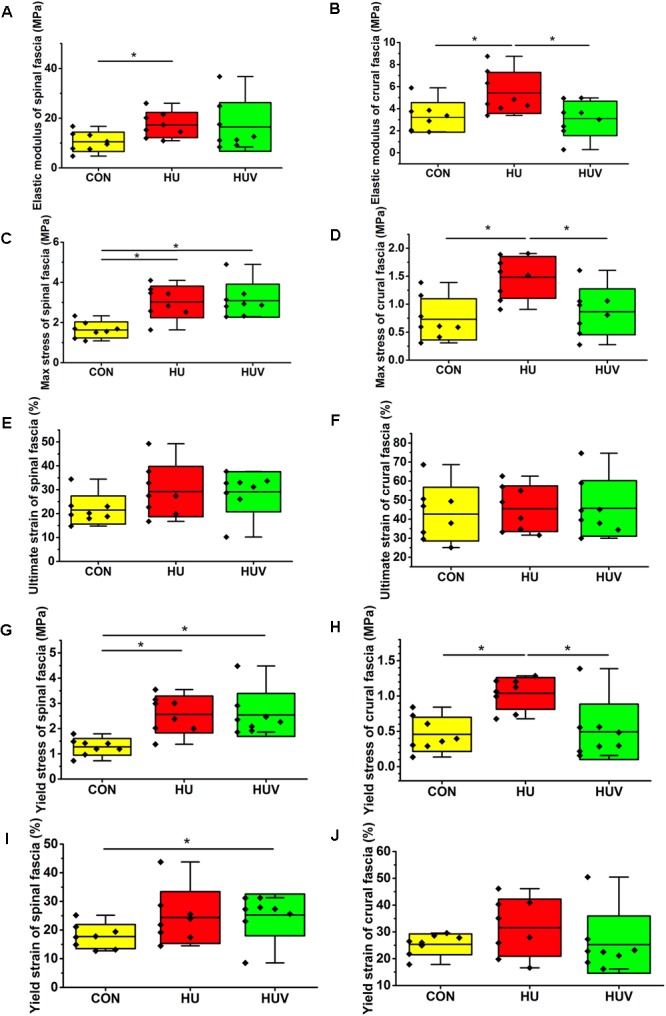
Biomechanical properties of fascia (*n* = 8). **(A)** Elastic modulus increased in spinal fascia in HU vs. CON. **(B)** Elastic modulus in crural fascia increased in HU vs. CON and HUV. **(C)** Maximal stress test of spinal fascia showed an increase in both HU and HUV vs. CON. **(D)** The maximal stress of crural fascia increased in HU vs. CON and HUV. **(E)** No differences in ultimate strain of spinal fascia among three groups. **(F)** No differences in ultimate strain of crural fascia. **(G)** Yield stress increased in spinal fascia in both HU and HUV vs. CON. **(H)** Yield stress of crural fascia showed an increase in HU vs. CON and HUV. **(I)** Yield strain of spinal fascia increased in HUV vs. CON. **(J)** No differences in yield strain of crural fascia. All groups are plotted as means = single horizontal black line in color bar; +/– standard deviation = top/bottom line of the box; max/min values = top/bottom of the bar line; Individual values were superimposed as dots. ^∗^*P* < 0.05.

In lumbar spinal fascia preparations, the elastic modulus (MPa = N/mm^2^, resistance to be deformed elastically) showed increased values in HU animals compared to CON (**Figure 3A**). The analysis of the maximal mechanical stress (MPa, resistance to fracture) of the spinal fascia preparations showed significant increased values in both HU and HUV rats compared to CON (**Figure 3C**). No differences were found in the ultimate strain (%) of spinal fascia among three groups (**Figure 3E**). The yield stress (MPa) increased in spinal fascia in both HU and HUV vs. CON (**Figure 3G**). The yield strain (%) of spinal fascia increased in HUV vs. CON (**Figure 3I**). Results show that the lumbar back fascia turned out to become mechanically stiffer following hindlimb unloading compared to CON animals. However, plantar vibration was not able to preserve normal mechanical properties of the spinal fascia between the two groups (HUV vs. HU).

#### Crural Fascia

The elastic modulus (MPa) as well as the max. stress (MPa) was found to be increased in HU in absence of vibration stimulation thus reflecting decreased crural fascia elasticity and increased resistance to tearing. Both parameters however remained largely unchanged in HUV in the presence of vibration vs. CON (**Figures 3B,D**). No differences were found in the ultimate strain (%) of crural fascia (**Figure 3F**). The yield stress (MPa) of crural fascia showed increase in HU vs. CON and HUV (**Figure 3H**). No differences were found in the yield strain (%) of crural fascia (**Figure 3J**). Results from the crural fascia confirmed that vibration mechano-stimulation in HUV rats showed robust effects *in vivo* by actually preserving close-to-normal mechanical properties in the calf region of the otherwise unloaded rat hindlimb.

### Qualitative and Quantitative Analysis of Deep Fascia

**FIGURE 4:**
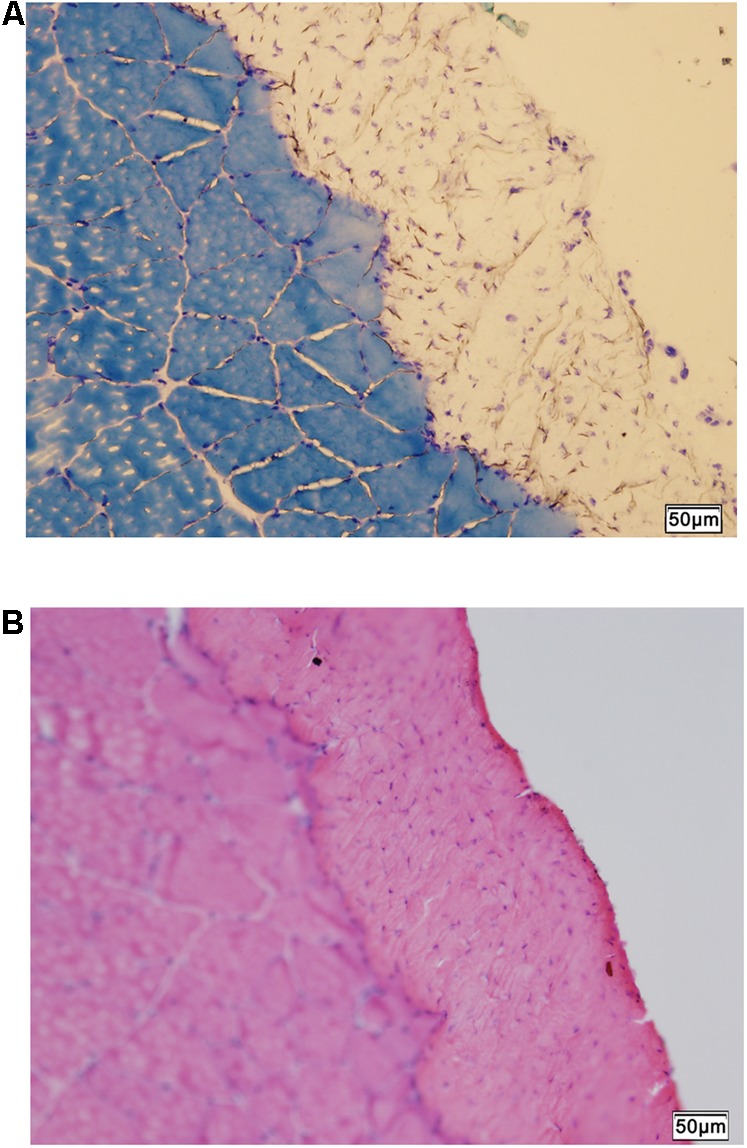
Histology of fascia. **(A)** Histologic staining by methylene blue staining shows skeletal muscle tissue supporting the overlying fascia (unstained). **(B)** Hematoxylin-eosin scout stain identifies the fascia tissue above skeletal muscle with cross-sectioned muscle fiber profiles. Bar = 50 μm

The normal histology of the CON rat crural fascia showed that fascia tissue layer was supported by the cross-sectioned profiles of skeletal muscle fiber bundles of muscle (**Figures 4A,B**).

#### Spinal Fascia

**FIGURE 5:**
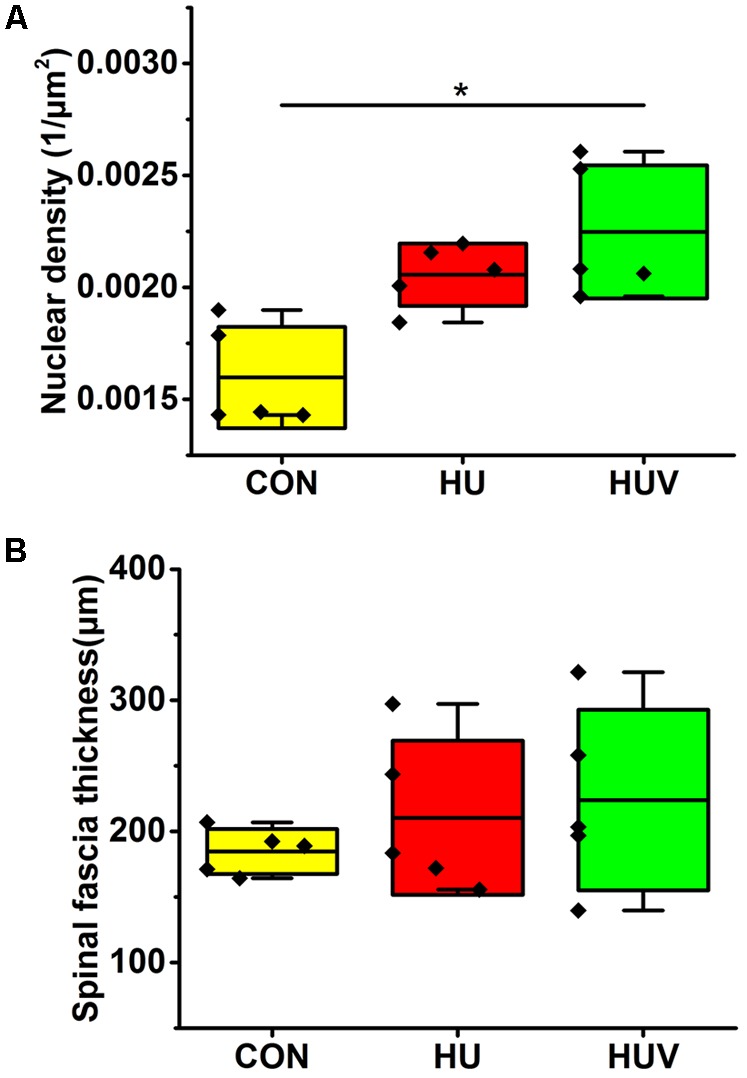
Morphometric analyses of spinal fascia (each, *n* = 5). **(A)** Nuclear density (DAPI fluorescence stained nuclei/μm^2^) increased in spinal fascia of HUV vs. CON. **(B)** Fascia thickness showed no changes in HU and HUV vs. CON. All groups are plotted as means = single horizontal black line in color bar; +/– standard deviation = top/bottom line of the box; max/min values = top/bottom of the bar line; Individual values were superimposed as dots. ^∗^*P* < 0.05.

**FIGURE 6:**
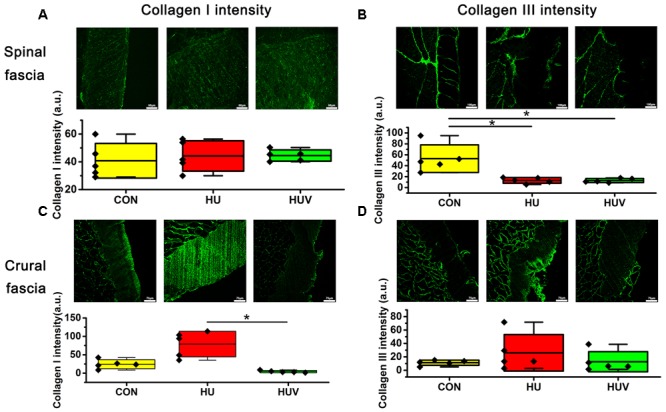
Immunohistochemistry of fascia (each, *n* = 5). **(A)** No differences of collagen I immunoreactivity in spinal fascia among three groups. Bar = 50 μm. **(B)** Collagen III immunoreactivity decreased in HU and HUV spinal fascia vs. CON. Bar = 100 μm. **(C)** Collagen I immunoreactivity increased in HU crural fascia vs. HUV. Bar = 75 μm. **(D)** No differences of collagen I immunoreactivity in crural fascia among three groups. Bar = 75 μm. a.u. is arbitrary units per unit area. All groups are plotted as means = single horizontal black line in color bar; + /– standard deviation = top/bottom line of the box; max/min values = top/bottom of the bar line; Individual values were superimposed as dots. ^∗^*P* < 0.05.

**FIGURE 7:**
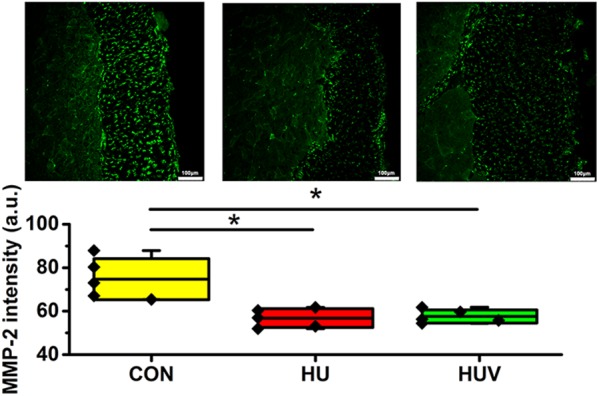
Matrix metalloproteinase-2 (MMP-2) immunoreactivity in fascia (each, *n* = 5). MMP-2 immunoreactivity reduced in HU and HUV spinal fascia vs. CON. a.u. is arbitrary units per unit area. All groups are plotted as means = single horizontal black line in color bar; +/– standard deviation = top/bottom line of the box; max/min values = top/bottom of the bar line; Individual values were superimposed as dots. Bar = 100 μm. ^∗^*P* < 0.05.

Compared to CON, the relative density of DAPI nuclei was only significantly increased in HUV animals (**Figure 5A**). Determination of the fascia morphometric dimension (cross-sectional thickness) showed no differences in either groups (HU, HUV) vs. CON (**Figure 5B**). In addition, intensity of collagen I immunoreactivity, an indirect marker of fascia stiffness, was similar between groups (**Figure 6A**). However, intensity of collagen III immunoreactivity, an indirect marker of tissue compliance, was decreased in both HU and HUV vs. CON thus confirming the altered mechanical properties found in HU and HUV animals after end of the unloading period (**Figure 6B**). We found an apparently stable size dimension of the spinal fascia tissue layer with increased nuclear density and decreased collagen III immunoreactivity patterns. In the spinal fascia MMP2 immunoreactivity, an indirect marker of connective tissue remodeling, was decreased in both HU and HUV vs. CON (**Figure 7**).

#### Crural Fascia

**FIGURE 8:**
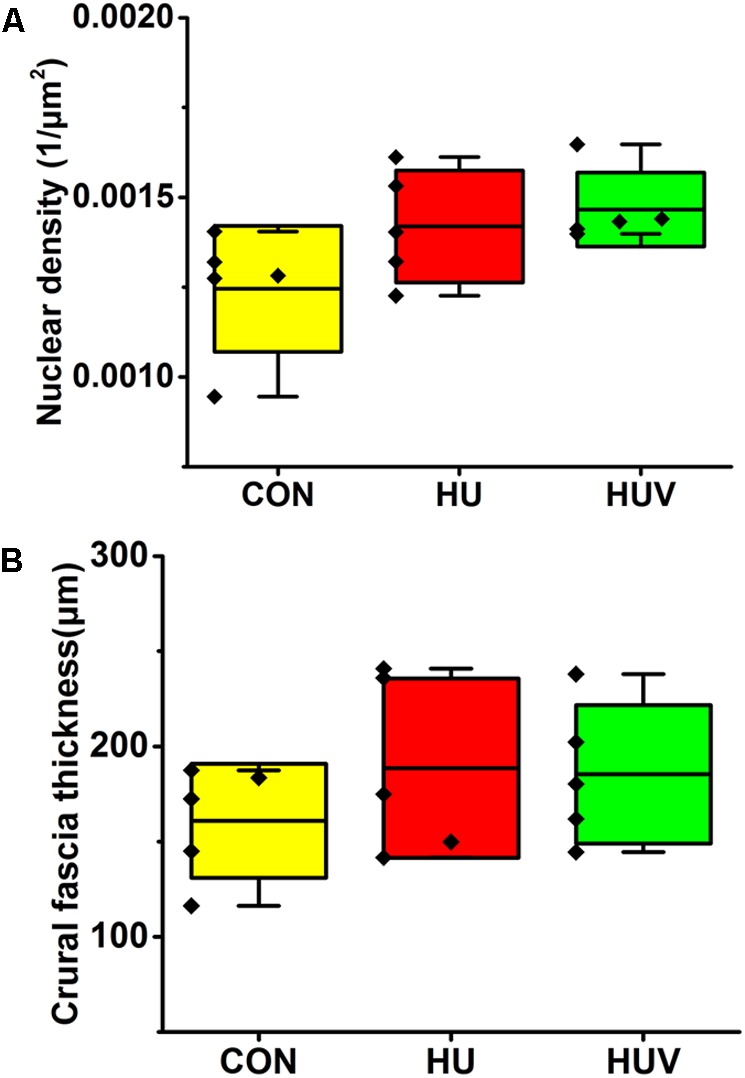
Morphometric analyses of crural fascia (each, *n* = 5). **(A)** No significant differences were found in nuclear density (DAPI fluorescence stained nuclei/μm^2^) in crural fascia. **(B)** Fascia thickness showed no changes in HU and HUV vs. CON. All groups are plotted as means = single horizontal black line in color bar; + /– standard deviation = top/bottom line of the box; max/min values = top/bottom of the bar line; Individual values were superimposed as dots.

No significant changes were found in the density of DAPI nuclei in crural fascia (**Figure 8A**). Determination of the fascia morphometric dimension (cross-sectional area) showed no differences in either groups (HU, HUV) vs. CON (**Figure 8B**). Intensity of collagen III immunoreactivity in the crural fascia was similar between groups (**Figure 6D**). However, collagen I antigen immunofluorescence intensity showed almost two times higher values (arbitrary units, a.u.) in HU rats (non-significant vs. CON) and decreased intensity levels in HUV vs. CON (**Figure 6C**).

## Discussion

The main study outcome suggested that plantar vibration stimulation was able to maintain tibial bone mineral density not seen in vertebrae spine and to prevent increased crural fascia stiffness with disproportionate collagen remodeling that was however found in the unloaded spinal fascia as well as in crural fascia in absence of vibration mechano-stimulation.

Consistent with previous studies (Alwood et al., 2010; Sun et al., 2013), results showed a significant bone loss in L3 vertebrae, femur and tibia of HU rats. The lack of mechanical stimulation, which is important for maintaining the bone function, could induce bone resorption and restrain bone formation (Zhang et al., 2008). Vibration could restrain bone resorption, promote bone formation, increase the amount of bone alkaline phosphatase and enhance muscle strength (Xie et al., 2006; Prisby et al., 2008). In the present study, local plantar vibration preserved BMD of tibia. The vibration mechanosignals loaded on hindlimbs of rats could improve the function of the musculoskeletal system, including bone, muscle and tendon (Kiiski et al., 2008; Sun et al., 2014). However, vibration in the present study had little or no effects on bone loss in vertebrae and femur. This might be explained by the fact that plantar vibration stimulation likely was compensated by the neuroreflexive activation of local muscle groups of the hip, knee and ankle joints (e.g., via co-contraction mechanisms) thereby minimizing the effects of vibration for, e.g., trunk muscle groups and related functionally linked fascia chains in the rat model of disuse used in the present study. Belavý et al. (2012) has found a reduction in lumbo-pelvic extensor and flexor co-contraction induced by resistive vibration exercise. The central nervous system would change the regulation of agonist and antagonist muscles to control movement or maintain body posture. This indicated that the effects of vibration on muscle might be reduced by decreased co-contraction.

Although the anatomical location and regular histologic structures of fascia have been previously studied (Kumka and Bonar, 2012), there are some knowledge gaps with respect to the peculiar microstructural and molecular regional composition, their roles in global and local movement control of the vertebrate body, or likewise in humans their functional roles in muscle tension or force production. In regard to more specific functions and roles of deep fascia, most of the biological mechanisms underlying biomechanics of the muscle-tendon-bone unit under normal conditions, disuse or exercise, are largely unknown. Type I and III collagens, the most abundant collagen fibers found in the fascia, play important roles in tissue biomechanical properties (Schleip et al., 2012). For example, collagen I is responsible for tissue stiffness and collagen III for tissue compliance (Érika et al., 2008). In this study, decreased collagen III and almost unchanged collagen I immunoreactivity in spinal fascia of HU largely coincided with the mechanical property outcome, decreased elasticity and increased tensile strength. In crural fascia of HU, increased collagen I and preservation of collagen III also confirmed the biomechanical parameter outcome, decreased elasticity and increased tensile strength. Collagen I immunoreactive fiber networks diffusely merged into deep fascia tissue separated by collagen III fiber bundles. The decreased elasticity and increased tensile strength in turn might be explained by the fact that under the condition of HU an altered structural composition of the collagen fiber network could be mainly responsible for tensile strength of skeletal muscle proposed earlier (Järvinen et al., 2002).

Fourteen days of rat hindlimb unloading showed a significant decrease in the soleus muscle mass and had almost no effect on tendon mass or expression of collagen I and III, transforming growth factor-β, and connective tissue growth factor in tendon (Kjaer et al., 2009). This suggesting there might be a compensation of decreased muscle force from other structures. Therefore in the present study, the increased stiffness and tensile strength in HU might reflect some compensatory mechanisms for decreased muscle force. The elasticity modulus of spinal fascia was much higher than seen in the crural fascia indicating that spinal fascia is working in a smaller range and needs to be stiffer than the fascia of the calf necessary to elicit higher power output. Crural fascia is apparently working in a broader functional range and needs to be more elastic to adapt to the calf muscle motions. As a result, an apparently stable size dimension of the spinal fascia tissue layer with increased nuclear density and decreased collagen III immunoreactive fiber deposits developed region specifically suggesting the presence of tissue remodeling mechanisms in disused spinal vs. crural fascia that needs further investigation.

Fascia tissue layers are likely affected by mechanical stimulation as well as through gene expression and collagen synthesis (Gorgey and Khalil, 2015). Fibroblasts, the major cell type in fascia, are highly adapted to local tissue environment and likely responsive to different mechanical stimulation (Kumka and Bonar, 2012). Mechanical loading or unloading of connective tissue likely induces expression of several growth factors, some are involved in collagen synthesis and turnover, including transforming growth factor-β, connective tissue growth factor, and insulin-like growth factor (Abrahamsson and Lohmander, 1996; Schild and Trueb, 2002). As a very special mechanical stimulation, vibration stimulation in general showed dramatic results in improving muscle tone and therefore increasing muscle strength (Delecluse et al., 2003).

In clinical settings, for example, vibration therapy applied on plantar fascia has been used to improve spasticity of lower limbs (Usuki and Tohyama, 2011). In our study, vibration locally applied to plantar hind paw region inhibited the increase of collagen I in crural fascia induced by HU. Notably, increase of stiffness and tensile strength induced by HU was both prevented by vibration. In other words, vibration mechano-stimulation was able to decrease collagen I networks to make fascia sheaths less stiffer for the benefit of more dynamic muscle motions.

There were some limitations in our study. Firstly, a wider range of vibration mode, testing on different age/sex of animals, and investigating more time points of HU could well have different outcome on fascia and should be investigated in future studies. Secondly, we showed only BMD results of bone since our main focus was on fascia in this work. However, similar experiments focused on bone were previously published (Sun et al., 2014). Thirdly, we did not examine the effect of vibration on normal control animals, which might be useful to explore the mechanism of how the vibration works on fascia. The present work however should be considered as explorative study of deep fascia, and further studies are needed to consolidate the results reported.

## Conclusion

Plantar vibration stimulation in the HU rat was able (i) to maintain tibial BMD not seen in vertebrae spine and (ii) to prevent increased crural fascia stiffness induced by disuse. Deep fascia could be a prime target for plantar vibration mechano-stimulation in the HU rat because of the parallel results of histology and biomechanical changes of spinal and crural fascia. The biomechanical as well as tissue and cell properties in crural fascia and quality of tibia bone (BMD) were preserved in the HUV rat suggesting common mechanisms in deep fascia and bone adaptation induced by local plantar vibration following disuse. This would imply that deep fascia changes might at least partly contribute to bone loss in HU. Furthermore, the present animal study provided novel ideas to help us in the design of more efficient countermeasures for better treatment of bone loss and muscle atrophy following extended disuse on the ground as well as in spaceflight.

## Author Contributions

DB and LS: conceptualization. YH and XY: data curation. YH and DB: formal analysis and writing – original draft. LS, DB, and YF: funding acquisition and validation. DB, YH, and LS: investigation. MS, YH, and XY: methodology. LS and YF: project administration and resources. LS: supervision. All authors: writing – review and editing.

## Conflict of Interest Statement

The authors declare that the research was conducted in the absence of any commercial or financial relationships that could be construed as a potential conflict of interest.

## Abbreviations

μCT: microcomputer tomography
BMD: bone mineral density
CON: cage-control
DAPI: fluorescent 4′, 6-diamidino-2-phenylindole
HU: hindlimb unloading
HUV: hindlimb unloading plus local vibration
MMP-2: matrix metalloproteinases-2
PBS: phosphate buffered saline solution
WBV: whole body vibration
